# Supplementary material for: High Antennal Expression of CYP6K1 and CYP4V2 Participate in the Recognition of Alarm Pheromones by Solenopsis invicta Buren
Source: Insects. 2025 Jan 5;16(1):43. doi: 10.3390/insects16010043 (PMC11765799; doi:10.3390/insects16010043)
Supplement: Supplementary file 1 [file insects-16-00043-s001.zip › Table S1-S3.pdf]

**Table S1.** RT-qPCR verification and analysis of gene and its specific primers

| Name | Gene ID | Forward primer | Reverse primer |
|---|---|---|---|
| *SinvCYP6k1* | LOC105199165 | CTGCATTGGTTCACGATTTG | GAGTCCGGGAATGAAGTTTG |
| *SinvCYP6k1-1* | LOC105197535 | CCTACAAGGTGCCAAATTCC | TCCGGATCGTATCTTTCTGG |
| *SinvCYP4C1* | LOC105202822 | AGTATGCCGCCAGAAGAACG | TAAACGGCTTGCCTCCCATC |
| *SinvCYP4C1-1* | LOC105202818 | AACATTCCCCAAGTCGACAG | TCCTTTTTCATGCGTTCCTC |
| *SinvCYP4C1-2* | LOC105202821 | GTTCGATCCAGATCGCTTTC | GCGAATCTCATGCCTACACA |
| *SinvCYP4V2* | LOC105202784 | TCGAGATGATGCTGAAAACG | TTGAATCAGTGGCCGTTAAA |
| *SinvGST1* | LOC105197561 | GCGAGAGCCTCGAATTTAC | CACAAAACGTTTCGAGAGAAAA |
| *SinvGST3* | LOC105202739 | GAAGCAGGGAAAACAGCAAG | AAAATCTTCGTTTAAAAAGCAACAA |
| *Circadian clock-controlled* | LOC105203866 | TGCAAACTTGCAAGCGTGATT | TTGATTTTCGGGATACCTGGG |
| *bulb-specific protein 3* | LOC105196156 | TCGTTACGAACGATAAATTGCGT | AGAGCTTCGGCAAAAATCTCTTTA |
| *ef1-β* | | CCTGAAGACCGATAAGGGCAT | GATTGTTGTGTTGGTGGTTTCC |

**Table S2.** Specific primers for PCR.

| Name | Gene ID | Forward primer) | Reverse primer |
|---|---|---|---|
| *SinvCYP6k1* | LOC105199165 | ATGGTACTTGTGGAATTAAT | TTATCGTGGTTCAATAGTCAG |
| *SinvCYP4V2* | LOC105202784 | ATGGTTTTTACAGTTGTATTAA | TTATAGATTCCGTTTTTCAATC |

**Table S3.** T7 promoter-specific primers.

| Name | Forward primer | Reverse primer |
|---|---|---|
| ds*SinvCYP6k1* | taatacgactcactatagggAACATTCGCTTGCCAAAGGG | taatacgactcactatagggGCACTGGAGTCCGGGAATG |
| ds*SinvCYP4V2* | taatacgactcactatagggGGGCTCACAGTCGAAAAGCA | taatacgactcactatagggACAGCTGATGCTGTGTCATC |
| ds*GFP* | taatacgactcactatagggGGAGAGGGTGAAGGTGATGC | taatacgactcactatagggCATAACCTTCGGGCATGGCA |
