# Supplementary material for: High Antennal Expression of CYP6K1 and CYP4V2 Participate in the Recognition of Alarm Pheromones by Solenopsis invicta Buren
Source: Insects. 2025 Jan 5;16(1):43. doi: 10.3390/insects16010043 (PMC11765799; doi:10.3390/insects16010043)
Supplement: Supplementary file 1 [file insects-16-00043-s001.zip › Table S5.pdf]

Table S5 All amino acid sequences of phylogenetic tree NCBI accession number.

Gene Name	Species	NCBI number
ApCYP315a1	*Acyrthosiphon pisum*	XP_001944183
ApCYP49a1	*Acyrthosiphon pisum*	XP_001946744
ApCYP301a1	*Acyrthosiphon pisum*	XP_001948959
SinvCYP6K1	*Solenopsis invicta*	XP_025986982.2
SinvCYP6K1-1	*Solenopsis invicta*	XP_039315612.1
SinvCYP4C1	*Solenopsis invicta*	XP_039312723.1
SinvCYP4C1-1	*Solenopsis invicta*	XP_011169812.1
SinvCYP4C1-2	*Solenopsis invicta*	XP_025990771.2
SinvCYP4V2	*Solenopsis invicta*	XP_011169761.2
LmigCYP301B1	*Locusta migratoria*	AVL92817.1
LmigCYP302A1	*Locusta migratoria*	AVL92818.1
LmigCYP314A1	*Locusta migratoria*	AVL92819.1
LmigCYP404D1	*Locusta migratoria*	AVL92826.1
ApCYP306a1	*Acyrthosiphon pisum*	XP_001947874
ApCYP15a1	*Acyrthosiphon pisum*	XP_001952620
ApCYP305a1	*Acyrthosiphon pisum*	XP_001950295
ApCYP307a1	*Acyrthosiphon pisum*	XP_001945761
ApCYP18a1	*Acyrthosiphon pisum*	XP_001947923
LmigCYP15H1	*Locusta migratoria*	AVL92814.1
LmigCYP15H2	*Locusta migratoria*	AVL92815.1
LmigCYP18A1	*Locusta migratoria*	AVL92816.1
LmigCYP303A1	*Locusta migratoria*	AVL92820.1
LmigCYP307A2	*Locusta migratoria*	AVL92854.1
DmelCYP4c3	*Drosophila melanogaster*	AAF57098.1
DmelCYP4g1	*Drosophila melanogaster*	NP_525031.1
DmelCYP4ae1	*Drosophila melanogaster*	NP_525044.1
DmelCYP4e3	*Drosophila melanogaster*	AAF52804.1
DmelCYP4p2	*Drosophila melanogaster*	AAF58963.1
DmelCYP4p1	*Drosophila melanogaster*	NP_524828.1
DmelCYP4p3	*Drosophila melanogaster*	NP_610473.1
DmelCYP313a4	*Drosophila melanogaster*	NP_650224.3
DmelCYP313a3	*Drosophila melanogaster*	NP_650170.2
DmelCYP9c1	*Drosophila melanogaster*	NP_523850.1
DmelCYP9b1	*Drosophila melanogaster*	NP_523645.1
DmelCYP9b2	*Drosophila melanogaster*	AAF59290.1
DmelCYP6a21	*Drosophila melanogaster*	NP_611003.2
DmelCYP6a9	*Drosophila melanogaster*	NP_523748.2
DmelCYP6a8	*Drosophila melanogaster*	NP_523749.2
DmelCYP6a2	*Drosophila melanogaster*	NP_523628.1

DmelCYP6a20	*Drosophila melanogaster*	NP_611002.2
AmelCYP314A1	*Apis mellifera*	NP_001035347.1
AmelCYP343A2	*Apis mellifera*	QIS68417.1
AmelCYP307a1	*Apis mellifera*	XP_026300894.1
AmelCYP4c3	*Apis mellifera*	XP_016767691.1
AmelCYP4C1	*Apis mellifera*	XP_006564430.1
AmelCYP6k1	*Apis mellifera*	XP_026302253.1
AmelCYP4G11	*Apis mellifera*	NP_001035323.1
SlitCYP4L4	*Spodoptera litura*	ARH52654.1
DhelCYP6BK18	*Dastarcus helophoroides*	AGJ51946.1
DhelCYP6BQ22	*Dastarcus helophoroides*	AGJ51945.1
DhelCYP6BQ21	*Dastarcus helophoroides*	AGJ51944.1
TcasCYP9AD1	*Tribolium castaneum*	EEZ99187.1
TcasCYP9AC1	*Tribolium castaneum*	EFA01242.2
TcasCYP9AF1	*Tribolium castaneum*	EFA09149.1
TcasCYP346A2	*Tribolium castaneum*	EFA04605.1
TcasCYP346A1	*Tribolium castaneum*	EFA04674.1
TcasCYP6BR3	*Tribolium castaneum*	EFA12627.1
TcasCYP6BR1	*Tribolium castaneum*	EFA12629.1
TcasCYP6BR2	*Tribolium castaneum*	EFA12628.1
TcasCYP6BS1	*Tribolium castaneum*	EEZ99243.2
TcasCYP6BQ12	*Tribolium castaneum*	EFA02825.1
TcasCYP6BQ6	*Tribolium castaneum*	EFA02820.1
TcasCYP6BQ7	*Tribolium castaneum*	EFA02821.2
TcasCYP6BQ10	*Tribolium castaneum*	EFA02823.1
TcasCYP6BQ11	*Tribolium castaneum*	EFA02824.1
Dpon345e2	*Dendroctonus ponderosae*	AFI45008.1
