# Supplementary figures and images for: High Antennal Expression of CYP6K1 and CYP4V2 Participate in the Recognition of Alarm Pheromones by Solenopsis invicta Buren
Source: Insects. 2025 Jan 5;16(1):43. doi: 10.3390/insects16010043 (PMC11765799; doi:10.3390/insects16010043)

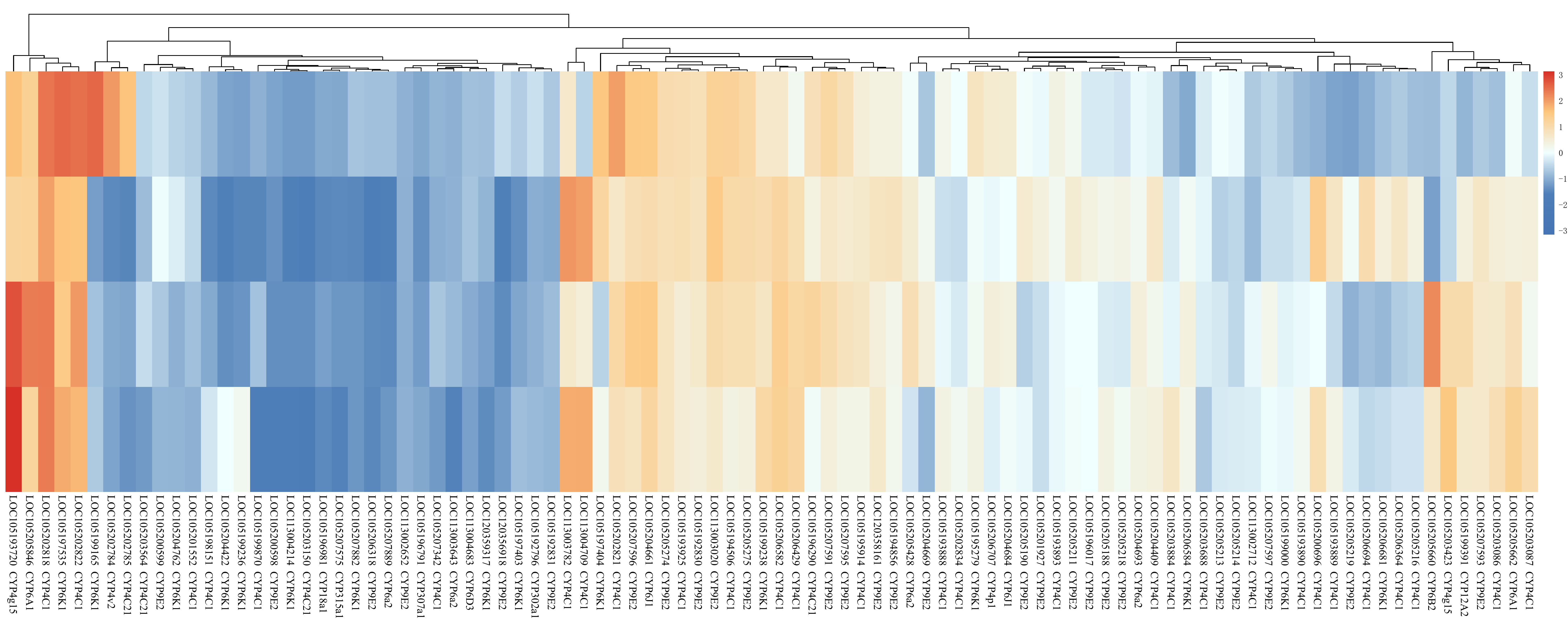

Supplement: Supplementary file 1 [file insects-16-00043-s001.zip › Fig S1.png]
